# The beneficial effects of menopausal hormone therapy on renal survival in postmenopausal Korean women from a nationwide health survey

**DOI:** 10.1038/s41598-021-93847-9

**Published:** 2021-07-29

**Authors:** Shin Young Ahn, Yoon Jin Choi, Jieun Kim, Gang Jee Ko, Young Joo Kwon, Kyungdo Han

**Affiliations:** 1 Department of Internal Medicine, Korea University College of Medicine, Seoul, Republic of Korea; 2 Department of Internal Medicine, Korea University Guro Hospital, Seoul, Republic of Korea; 3 Department of Internal Medicine, Yonsei University College of Medicine, Seoul, Republic of Korea; 4 Department of Statistics and Actuarial Science, Soongsil University, 369 Sangdo-ro, Dongjak-gu, Seoul, 06978 Republic of Korea

**Keywords:** Kidney diseases, Medical research, Epidemiology

## Abstract

Several studies have demonstrated the nephroprotective effects of estrogen on renal damage. In light of the inconsistent results of previous findings, this study aims to evaluate the in-depth role of menopausal hormone therapy (MHT) on the development of end stage renal disease (ESRD). 3,109,506 Korean adult women who had undergone a medical examination in 2009 (index year) were initially identified for inclusion in this study. We excluded subjects had not experienced menopause naturally, had data missing for at least one variable, and were diagnosed with ESRD within 1 year from the index year. MHT data was obtained from self-reporting questionnaires and the primary outcome was the development of ESRD from the index year until December 31, 2018. A final total of 1,460,311 subjects were included in this study. The participants were divided into four groups according to the duration of MHT; no history of MHT, MHT < 2 years, 2 ≤ MHT < 5 years, MHT ≥ 5 years. During the 9-year study period, a total of 4905 participants developed ESRD. The participants who had a history of MHT use were found to have a 30% reduced risk of developing ESRD. Results from the subgroup analyses were similar to that of the primary study. The findings in this study demonstrate the beneficial effects of MHT on the development of ESRD in postmenopausal women. Based on results, our study may offer suggestions for further studies to investigate the therapeutic options on kidney disease.

## Introduction

A significant factor for explaining the differences in disease incidence and prevalence is biological sex. Cardiovascular disease (CVD), considered a representative disease, highlights the important differences between morbidity and mortality risk in men and women^1^. Throughout previous studies, sex hormones have been shown to have an important role in the development and progression of various CVDs^2^. Similar sex disparities have been reported in kidney diseases including incidence, prevalence, rate of progression, and outcomes of kidney diseases. Furthermore, several factors related to sex, such as genetics, hormones, social habits, economic status, and family support affect the development, progression, burden, and outcome of renal disease^3,4^. Despite the global burden of chronic kidney disease (CKD), little attention has been given to sex disparities in CKD. Moreover, the results of studies that explore the implications of sex hormones on kidney diseases are still inconsistent^5–7^ and mainly male versus female.

The beneficial effects of estrogen on the kidney have been well explored through many clinical^8,9^ and experimental studies^10,11^. As expected, a peak in the prevalence of end stage renal disease (ESRD) was observed in perimenopausal women, when the activity of female hormones started to decline^12^. However, there are few studies that investigate the benefits of estrogen treatment on the development and progression of kidney disease.

Therefore, this paper further investigates the beneficial effects of menopausal hormone therapy (MHT) on the development of ESRD in postmenopausal women by significantly expanding the population size through the utilization of a nationwide population-based study. An overall improved understanding of the effects of MHT can better help nephrologists to address patient needs.

## Results

### Baseline characteristics of study population

The study population was assigned to four groups according to the duration of MHT; no history of MHT, MHT < 2 years, 2 ≤ MHT < 5 years, MHT ≥ 5 years. 84.1% (n = 1,228,203 participants) of the study population had not received MHT compared with 15.9% (n = 232,108 participants) who had. The baseline characteristics of the cohort are shown in Table 1. The subjects with no history of MHT were more likely to be older and have diabetes, hypertension, dyslipidemia, CKD, and obesity. As expected, they were less likely to smoke, drink alcohol, or exercise.

**Table 1:** Baseline characteristics of the study populations according to history of MHT.

Variables	No MHT (n = 1,228,203)	MHT			*p-*value
		< 2 years (n = 134,394)	2 ≤ < 5 years (n = 55,187)	≥ 5 years (n = 42,527)	
Age	62.5 ± 8.7	58.4 ± 6.7	59.9 ± 6.4	60.8 ± 6.0	< 0.0001
**Age groups**					< 0.0001
40 ≤ age < 50	37,597 (3.1%)	8070 (6%)	2353 (4.3%)	979 (2.3%)	
50 ≤ age < 60	462,074 (37.6%)	69,613 (51.8%)	27,657 (50.1%)	15,711 (36.9%)	
Age ≥ 60 years	728,532 (59.3%)	56,711 (42.2%)	25,177 (45.6%)	25,837 (60.8%)	
Diabetes	172,124 (14.0%)	13,527 (10.1%)	5157 (9.3%)	4330 (10.2%)	< 0.0001
Hypertension	550,614 (44.8%)	47,804 (35.6%)	20,020 (36.3%)	16,951 (39.9%)	< 0.0001
Dyslipidemia	421,498 (34.3%)	46,087 (34.3%)	17,983 (32.6%)	13,533 (31.8%)	< 0.0001
CKD	154,623 (12.6%)	12,347 (9.2%)	5442 (9.9%)	5352 (12.6%)	< 0.0001
WC (cm)	80.4 ± 8.6	78.8 ± 8.1	78.2 ± 8.5	78.4 ± 7.6	< 0.0001
BMI (kg/m^2^)	24.3 ± 3.2	23.9 ± 2.9	23.7 ± 2.8	23.8 ± 2.8	< 0.0001
Obesity (BMI ≥ 25 kg/m^2^)	472,861 (38.5%)	44,442 (33.1%)	16,382 (29.7%)	13,288 (31.3%)	< 0.0001
**Smoking status**					< 0.0001
Never smoker	1,183,715 (96.4%)	128,411 (95.6%)	52,601 (95.3%)	40,538 (95.3%)	
Ex-smoker	12,002 (1.0%)	1991 (1.5%)	894 (1.6%)	747 (1.8%)	
Current smoker	32,486 (2.7%)	3992 (3.0%)	1692 (3.1%)	1242 (2.9%)	
**Alcohol consumption**					< 0.0001
No.	1,087,587 (88.6%)	112,517 (83.7%)	45,919 (83.2%)	35,886 (84.4%)	
Mild drink	134,837 (11.0%)	20,977 (15.6%)	8890 (16.1%)	6359 (15.0%)	
Heavy drink	5779 (0.5%)	900 (0.7%)	378 (0.7%)	282 (0.7%)	
Physical activity	210,843 (17.2%)	30,161 (22.5%)	13,487 (24.4%)	11,190 (26.3%)	< 0.0001

*BMI* body mass index, *CKD* chronic kidney disease, *MHT* menopausal hormone therapy, *WC* waist circumference.

P < 0.05 indicates a significant difference among groups by ANOVA.

The reproductive histories of the four groups of women are shown in Table 2. The participants who had never received MHT were more likely to have more than two offspring, a longer duration of breast feeding (≥ 12 months), and to have started menstruation at an older age. Additional findings showed that they were less likely to take oral contraceptives and that the percentage experiencing menopause before the age of fifty was low.

**Table 2:** Reproductive parameters of the study population according to history of MHT.

Variables	No MHT (n = 1,228,203)	MHT			*p-*value
		< 2 years (n = 134,394)	2 ≤ < 5 years (n = 55,187)	≥ 5 years (n = 42,527)	
**Parity**					< 0.0001
1 person	66,774 (5.4%)	10,996 (8.2%)	4788 (8.7%)	3461 (8.1%)	
≥ 2 persons	1,135,472 (92.5%)	118,525 (88.2%)	48,320 (87.6%)	37,254 (87.6%)	
No	25,957 (2.1%)	11,957 (2.9%)	4973 (3.2%)	4407 (3.5%)	
**Breast feeding**					< 0.0001
< 6 months	71,822 (5.9%)	11,527 (8.6%)	4822 (8.7%)	3338 (7.9%)	
6 ≤ < 12 months	200,851 (16.4%)	27,349 (20.4%)	11,459 (20.8%)	7548 (17.8%)	
≥ 12 months	883,625 (71.9%)	83,960 (62.5%)	33,630 (60.9%)	27,583 (64.9%)	
Not done	71,905 (5.9%)	11,558 (8.6%)	5276 (9.6%)	4058 (9.5%)	
**Oral contraceptives**					< 0.0001
No	1,023,263 (83.3%)	96,284 (71.6%)	39,055 (70.8%)	29,619 (69.7%)	
Yes	164,257 (13.4%)	32,866 (24.5%)	13,450 (24.4%)	10,560 (24.8%)	
Unknown	40,683 (3.3%)	5,244 (3.9%)	2,682 (4.9%)	2,348 (5.5%)	
Age at menarche	16.5 ± 1.8	16.2 ± 1.8	16.2 ± 1.8	16.3 ± 1.8	< 0.0001
Menarche < 11 years	893 (0.1%)	126 (0.1%)	45 (0.1%)	31 (0.1%)	0.0573
Age at menopause	50.0 ± 4.0	50.0 ± 4.0	50.0 ± 4.2	49.4 ± 4.8	< 0.0001
**Groups of menopause ages**					< 0.0001
< 50 years	418,092 (34.0%)	50,630 (37.7%)	21,590 (39.1%)	18,828 (44.3%)	
50 ≤ age < 55 years	679,630 (55.3%)	69,609 (51.8%)	27,173 (49.3%)	18,658 (43.9%)	
≥ 55 years	130,481 (10.6%)	14,155 (10.5%)	6,424 (11.6%)	5,041 (11.9%)	
Follow up period (years)	9.2 ± 1.3	9.3 ± 0.8	9.3 ± 0.8	9.3 ± 0.8	< 0.0001

*MHT* menopausal hormone therapy.

P < 0.05 indicates a significant difference among groups by ANOVA.

### MHT and the risk of ESRD

**Figure 1:**
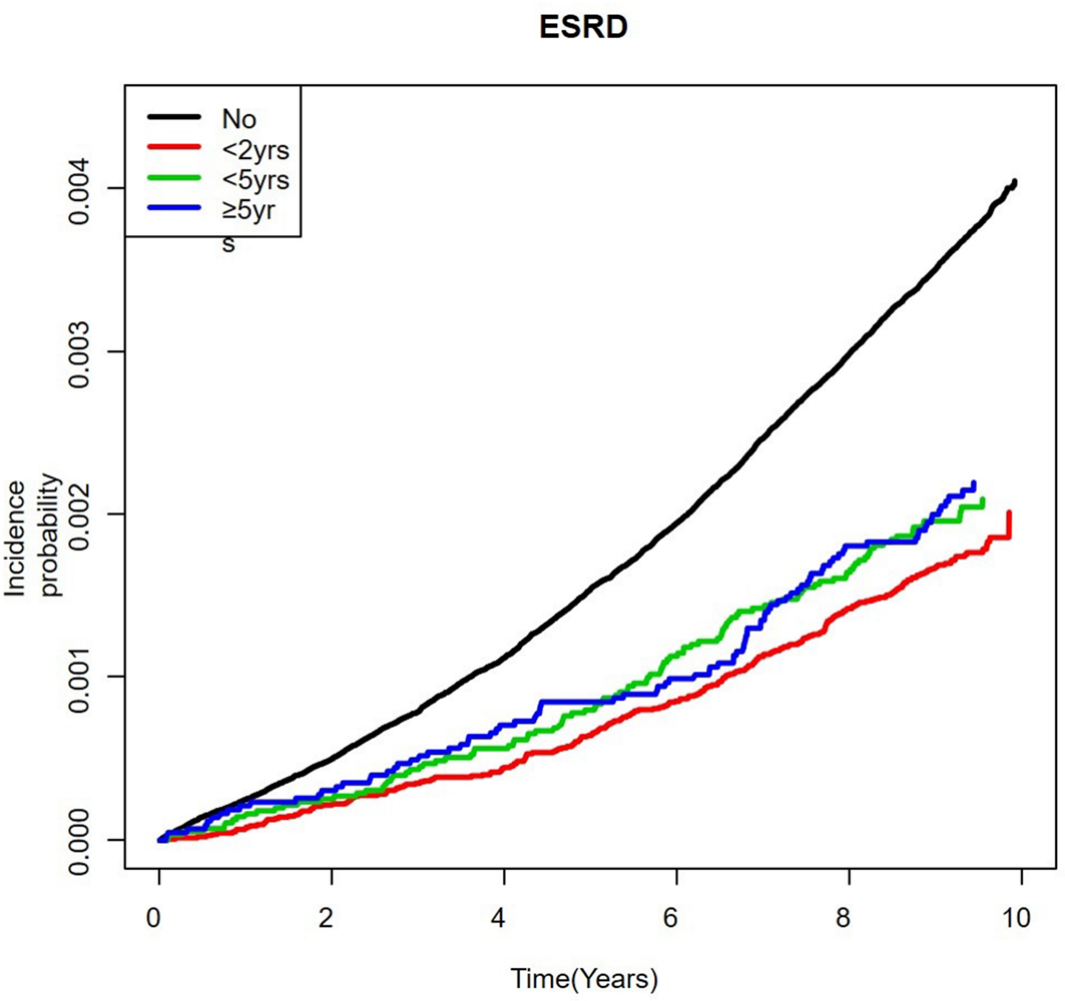
The Kaplan–Meier curves for development of ESRD according to the duration of MHT.

During the 9-year study period, a total of 4,905 participants developed ESRD. The Kaplan–Meier curves showed a significant overall reduction in the incidence of ESRD in subjects who had received MHT regardless of duration (Fig. 1). After adjusting for possible confounding factors such as age, BMI, smoking history, alcohol intake, physical activities, hypertension, diabetes, dyslipidemia, and CKD, the participants who had a history of MHT use had a significantly lower risk of developing ESRD (MHT < 2 years, HR 0.634, 95% CI 0.556–0.723; 2 ≤ MHT < 5 years, HR 0.721, 95% CI 0.597–0.871; MHT ≥ 5 years, HR 0.654, 95% CI 0.531–0.806) (Table 3).

**Table 3:** Hazard ratios and 95% confidence intervals of ESRD development by history of MHT.

Total population	No.	Outcome	IR	Model 1^a^	Model 2^b^
**MHT**					
No MHT	1,228,203	4468	0.397	1 (ref.)	1 (ref.)
MHT < 2 years	134,394	236	0.188	0.474 (0.415, 0.540)	0.634 (0.556, 0.723)
2 ≤ MHT < 5 years	55,187	111	0.216	0.543 (0.450, 0.655)	0.721 (0.597, 0.871)
MHT ≥ 5 years	42,527	90	0.227	0.571 (0.463, 0.703)	0.654 (0.531, 0.806)

*ESRD* end stage renal disease, *MHT* menopausal hormone therapy.

^a^Model 1: Not adjusted.

^b^Model 2: Cox proportional model adjusted for age, BMI, smoking history, drinking history, degree of exercise, hypertension, dyslipidemia, diabetes, and CKD.

Our research also showed that the early menopausal group (age at a menopause < 50 years) had an increased risk for the incidence of ESRD (HR 1.079, 95% CI 1.016–1.146) compared to the reference group (50 ≤ age at menopause < 55 years) (Table S1).

### Subgroup analysis

Stratified analysis according to age, diabetes, hypertension, obesity and CKD revealed further patterns in the data (Table 4). Subjects receiving MHT consistently showed a reduced risk of ESRD development compared to subjects without MHT. In particular, the beneficial effects of MHT were clearly observed in participants who were younger (< 65 years), had diabetes and hypertension. These favorable effects of MHT continued regardless of obesity and CKD status (Table 4).

**Table 4:** Hazard ratios and 95% confidence intervals of ESRD development by history of MHT in subgroups.

Subgroups	No.	Outcome	IR	HR (95% CI)*
**Age < 65 years**				
No MHT	744,473	1900	0.274	1 (ref.)
MHT < 2 years	110,403	155	0.150	0.633 (0.538, 0.746)
2 ≤ MHT < 5 years	45,591	75	0.176	0.735 (0.583, 0.926)
MHT ≥ 5 years	32,121	52	0.173	0.639 (0.485, 0.843)
**Age ≥ 65 years**				
No MHT	483,730	2568	0.594	1 (ref.)
MHT < 2 years	23,991	81	0.367	0.660 (0.528, 0.824)
2 ≤ MHT < 5 years	9596	36	0.408	0.740 (0.532, 1.029)
MHT ≥ 5 years	10,406	38	0.395	0.710 (0.515, 0.979)
**Diabetes (−)**				
No MHT	1,056,079	2231	0.229	1 (ref.)
MHT < 2 years	120,867	140	0.124	0.693 (0.584, 0.824)
2 ≤ MHT < 5 years	50,030	75	0.161	0.858 (0.680, 1.081)
MHT ≥ 5 years	38,197	53	0.149	0.664 (0.505, 0.872)
**Diabetes (+)**				
No MHT	172,124	2237	1.455	1 (ref.)
MHT < 2 years	13,527	96	0.769	0.578 (0.471, 0.710)
2 ≤ MHT < 5 years	5157	36	0.760	0.560 (0.403, 0.779)
MHT ≥ 5 years	4330	37	0.926	0.647 (0.467, 0.895)
**Hypertension (−)**				
No MHT	677,589	796	0.127	1 (ref.)
MHT < 2 years	86,590	45	0.056	0.555 (0.410, 0.752)
2 ≤ MHT < 5 years	35,167	29	0.088	0.860 (0.593, 1.248)
MHT ≥ 5 years	25,576	17	0.071	0.611 (0.378, 0.988)
**Hypertension (+)**				
No MHT	550,614	3672	0.735	1 (ref.)
MHT < 2 years	47,804	191	0.430	0.662 (0.572, 0.767)
2 ≤ MHT < 5 years	20,020	82	0.441	0.686 (0.550, 0.854)
MHT ≥ 5 years	16,951	73	0.464	0.660 (0.523, 0.833)
**BMI < 25 kg/m**^**2**^				
No MHT	755,342	2478	0.359	1 (ref.)
MHT < 2 years	89,952	143	0.170	0.644 (0.543, 0.763)
2 ≤ MHT < 5 years	38,805	73	0.202	0.746 (0.591, 0.943)
MHT ≥ 5 years	29,239	59	0.216	0.670 (0.517, 0.868)
**BMI ≥ 25 kg/m**^**2**^				
No MHT	472,861	1990	0.457	1 (ref.)
MHT < 2 years	44,442	93	0.224	0.631 (0.512, 0.777)
2 ≤ MHT < 5 years	16,382	38	0.249	0.695 (0.504, 0.958)
MHT ≥ 5 years	13,288	31	0.250	0.651 (0.456, 0.929)
**CKD (−)**				
No MHT	1,073,580	1523	0.15384	1 (ref.)
MHT < 2 years	122,047	92	0.08073	0.675 (0.546, 0.835)
2 ≤ MHT < 5 years	49,745	38	0.08182	0.698 (0.506, 0.965)
MHT ≥ 5 years	37,175	30	0.08642	0.679 (0.473, 0.975)
**CKD (+)**				
No MHT	154,623	2945	2.161	1 (ref.)
MHT < 2 years	12,347	144	1.268	0.610 (0.515, 0.721)
2 ≤ MHT < 5 years	5442	73	1.461	0.725 (0.574, 0.915)
MHT ≥ 5 years	5352	60	1.218	0.622 (0.482, 0.804)

*ESRD* end stage renal disease, *MHT* menopausal hormone therapy, *BMI*, body mass index, *CKD*, chronic kidney disease.

*HR Cox proportional model adjusted for age, BMI, smoking history, drinking history, degree of exercise, hypertension, dyslipidemia, diabetes, and CKD.

## Discussion

This nationwide population-based study showed the beneficial effects of MHT on the development of ESRD in postmenopausal women during the 9-year study period. MHT significantly reduced the risk of developing ESRD regardless of duration. These favorable MHT effects confirmed even after adjusting for potential confounders including age, BMI, lifestyle, and comorbidities. In addition, stratified analyses were performed in subgroups based on age, diabetes, hypertension, obesity, and CKD status. As with the original findings in this study, MHT was also shown to have reduced the risk of incident ESRD in almost all stratified subgroups. Finally, we observed that the age for menopause might affect the risk of ESRD development. As expected, the younger onset of menopause showed a higher risk of ESRD incidence even after multivariate adjustments. These results may be evidence that a female hormone, represented by estrogen, may help slow the progression of ESRD.

In previous studies, we found clear and consistent sex disparities in the clinical course and outcomes of patient with CKD^13–16^. Clinical and experimental research has demonstrated the protective effect of estrogen on renal outcome. The findings showed that estrogen provides a protective effect on the physiologic and pathologic processes of the kidney via the three estrogen receptors located in the renal tissues^17–19^. The animal models showed that the antifibrotic and anti-apoptotic effects of estrogen retard the rate of CKD progression in diabetic^20^ and nondiabetic nephropathy. Specifically, models included aging^21^, polycystic disease^22^, ischemic-reperfusion injury^23^, and adenine treatment^24^. Through histologic evidences of those experimental models, we demonstrated that estrogen reduced mesangial expansion, attenuated the glomerular hypertrophy as well as podocyte loss, and decreased interstitial fibrosis and collagen deposition. In contrast, male sex hormones are known to have deleterious effects that contribute to inflammatory process in the physiologic and pathologic conditions. In diabetic castrated male rats, several hallmarks of kidney damages such as, albuminuria, glomerulosclerosis, and tubulointerstitial fibrosis worsened in proportion to the dose of dihydrotestosterone^25^. Testosterone supplementation also induced podocyte apoptosis in ovariectomized wild-type mice^26^. In various other disease models, testosterone had a negative effect on kidney damage^24,27^. Based on these findings, it can be deduced that estrogen provides a renoprotective effect for women. However, despite numerous published studies on the link between sex hormones and CKD, the effects are still controversial.

In general, population based studies reveal that the prevalence of CKD among women is higher than in men. Additionally, epidemiologic studies further support this finding^13^. There are several explanations for these results. First, women have longer life expectancy, as a result, aging process can cause decline of kidney function naturally. Second, the equations for estimating GFR, such as MDRD or Chronic Kidney Disease Epidemiology Collaboration (CKD-EPI) have several measurements bias. These then lead to underestimated eGFR calculations by these equations in women^28,29^. Therefore, sex-specific thresholds of eGFR are required to determine an accurate prevalence measurement of CKD^30^.

Although, the overall prevalence of CKD is higher in women and the CKD progression differences disappear in postmenopausal women, the rate of CKD progression is lower in premenopausal women than in men of the same age^31^. This phenomenon has been explained by the results of the aforementioned studies. Moreover, most population-based studies demonstrate that men have a steeper slope of the rate of eGFR decline than women^32^. The rate of eGFR decline has been attributed to the contrary effects of sex hormones, comparatively higher prevalence of unhealthy lifestyles in men, and the inherent protective effects of estrogen^33–35^. In addition to these differences, we also observed the beneficial effects of estrogen therapy on the development of ESRD in postmenopausal women. In connection to this observation, we showed that women who experience an earlier onset of menopause, meaning a shorter period of estrogen production, yielded a faster progression of ESRD. Our results are consistent with the results of previous studies showing the protective role of estrogen in the development and progression of CKD.

There are limitations to our study. First, the data regarding reproductive history were collected by self-reporting questionnaires (Supplementary Material). Therefore, there was also inevitably a recall bias and we could not obtain detailed information about MHT; drug formulation, routes of administration, compliance over the duration of MHT, and reasons for stopping MHT. However, menopausal women who did not experience menopause naturally because of hysterectomy or bilateral oophorectomy were excluded from the analysis in this study. Considering that the physicians prescribed an estrogen-progestogen combination drug to women with the uterus because of the risk of endometrial cancer, the MHT drug used in this study is more likely to be a combination drug. According to an analysis of the domestic consumption of postmenopausal hormonal medicine in the year 2010 reported by Cho et al., combination drug was the highest at 53%, followed by Tibolone at 40% and estrogen alone at 7%^36^. Second, this was a population-based observational study. Therefore, there might be confounders that we did not consider. Third, the participants who received hormone therapy were more likely to have an interest in their health and well-being and they have a healthier lifestyle which contributes to slow development and progression of CKD. Fourth, the study population consisted of Korean postmenopausal women and so far that reason, the results of this study could not be generalized to other ethnicities. However, we can find other population studies that are based on various ethnicities and demonstrated the protective effect of estrogen on renal outcome. Fifth, the causes of ESRD were not identified in our study.

Despite the limitations mentioned above, our study is the largest population-based study to demonstrate the favorable effects of MHT on the development of ESRD in postmenopausal women. This study analyzed a 9-year period of data contained in the Korean NHID that includes medical information for 50 million Koreans making it sufficient to overcome several unmeasured biases.

Our study showed the presence of beneficial effects of MHT on the development of ESRD in postmenopausal women during the 9-year study period. MHT, regardless of duration, significantly reduced the risk of developing ESRD. These favorable effects were confirmed even after conducting subgroup analyses. In existing literature, there is a still controversy for the effect of sex hormones on the development and progression of kidney disease. However, this study finds that female hormones based on estrogen provides a protective effect against ESRD. Therefore, our study may offer suggestions for further studies to investigate the therapeutic effects of estrogen on kidney disease.

## Methods

### Data source and study population

The National Health Insurance Service (NHIS) of Korea provided access to the National Health Insurance Database (NHID) that contains data on eligibility, medical treatment, health examination, and medical care institution^37,38^. The NHID contains the health information of approximately 50 million Koreans who are enrolled in a single-payer healthcare system and are required to have biannual standardized medical examinations.

**Figure 2:**
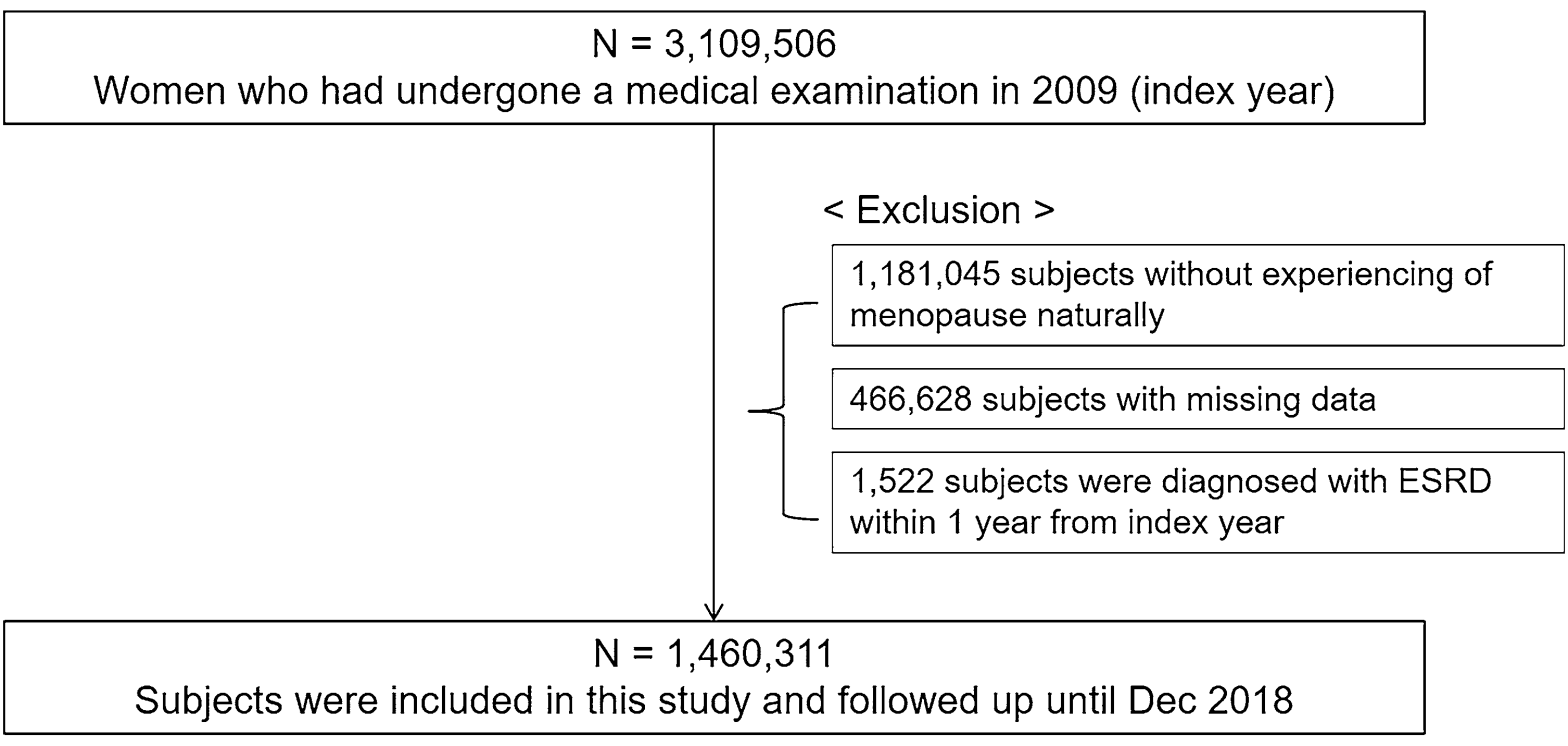
The schematic flow chart of the study population.

3,109,506 Korean adult women who had undergone a medical examination in 2009 (index year) were initially identified for inclusion in this study. We excluded subjects who were diagnosed as not having menopause naturally (n = 1,181,045), had missing data for at least one variable (n = 466,628), and were diagnosed with ESRD within 1 year from the index year (n = 1,522) (Fig. 2). As a consequence, 1,460,311 subjects were included in this study and tracked from the index year to December 31, 2018.

### Ethics declarations

This study was approved by the Institutional Review Board of the Korea University Guro Hospital. (IRB No. 2019GR0222). This study was also approved by National Health Insurance Service (NHIS-2020-1-307). The informed consent was waived for all subjects by the Committee of Institutional Review Board of the Korea University Guro Hospital.

### Demographic and lifestyle variables

Demographic characteristics including age, gender, amount of alcohol consumption, smoking status, and physical activity were collected using self-reported questionnaires. Medical examination data included height, weight, waist circumference, blood pressure, laboratory tests, past medical history, and health related behaviors. Definitions of demographic and lifestyle variables are described in accordance to previous studies^39,40^. Participants were categorized into 3 groups based on drinking frequency: non-drinker, ≤ 1 drink per month; light drinker, > 1 drink but < 2 drinks per week; heavy drinker, > 2 drinks per week. The study population was also assigned to one of 3 groups according to the participant’s smoking status: non-smoker, ex-smoker, and smoker. A “yes” response for the physical activity query required the participant to be active beyond the normal activities of daily living, that the duration exceeds 30 min, and have a frequency of more than five times per week.

### Variables for reproduction

The questionnaires yielded data for the variables for reproduction (Supplementary Material): age at menarche, age at menopause, parity number, history of oral contraceptives, history of MHT, and history of breast feeding. Menopause was defined as the absence of menstruations for 12 consecutive months in the absence of procedures that stopped menstruation including hysterectomy and oophorectomy. Study populations were categorized into 4 groups differentiated based on the duration of MHT; no MHT, MHT < 2 years, 2 ≤ MHT < 5 years, and MHT ≥ 5 years. Parity was defined in terms live births and divided into 3 groups; no live birth, one live birth, and more than two live births. Participants were also categorized into 4 groups according to the duration of breast feeding; no breast feeding, breast feeding < 6 months, 6 ≤ breast feeding < 12 months, and breast feeding ≥ 12 months. We also divided the study populations into several subgroups depending on age at menarche (age < 11 years and age ≥ 11 years) and age at menopause (age < 50 years, 50 ≤ age < 55 years, and age ≥ 55 years).

### Comorbidities

The detailed definitions of comorbidities are described in previous studies^41^. Patients with hypertension included those who submitted either at least one claim in a year for a prescription of anti-hypertensive medication or measured systolic/diastolic blood pressure ≥ 140/90 mmHg. Diabetes included those who submitted at least one claim in a year for a prescription of anti-diabetic medication or fasting plasma glucose level ≥ 126 mg/dL. Lastly, dyslipidemia included those who submitted either at least one claim in a year for a prescription of anti-dyslipidemic medication or exhibited a total serum cholesterol level ≥ 240 mg/dL. Glomerular filtration rate (GFR) was calculated using the four variable isotope dilution mass spectrometry (IDMS) Modification of Diet in Renal Disease (MDRD) formula: 175 × serum Cr (mg/dL)^−1.154^ × age (year)^−0.203^ × 0.742 (if female)^42^ and CKD was defined as an eGFR < 60 mL/min/1.73 m^2^^43^. Body-mass index (BMI) and waist circumference data were measured by a trained examiner. Obesity was defined as a BMI of more than 25 kg/m^2^ according to the Korean Endocrine Society^44^. According to the World Health Organization (WHO), the cutoff value for defining obesity is lower for East Asians than for non-Asians.

### Primary outcome

The primary outcome was incident ESRD until December 31, 2018. ESRD was defined using the combination of a V code assigned to patients who required hemodialysis (V001), peritoneal dialysis (V003), or kidney transplantation (V005) and an ICD-10 code (N18-19, Z49, Z94.0, Z99.2), as previously defined in other studies^41^. All medical costs for dialysis were reimbursed and ESRD patients were registered as special medical aid beneficiaries. This enabled the location of all ESRD patients in the Korean population, especially data on those who had started dialysis. However, we excluded participants with no previous chronic kidney disease who had a kidney transplantation or dialysis code on the same date as an acute renal failure code. Subjects receiving continuous renal replacement therapy or acute peritoneal dialysis were also excluded.

### Statistical analysis

Data are presented as the mean ± standard deviation for continuous variables and the numbers and percentages for categorical variables. The baseline demographic, lifestyle, and reproductive variables were compared according to subgroups based on history and duration of MHT using ANOVA test for continuous variables and chi-squared test for categorical variables. The cumulative incidence of ESRD according to the duration of MHT was evaluated using the Kaplan–Meier curves, and log-rank test was used to analyze differences between groups. The multivariable-adjusted proportional-hazards model was applied. It was adjusted for age, BMI, smoking status, categories of drinking frequency, physical activity, HTN, DM, dyslipidemia, and CKD. The hazard ratios (HRs) and 95% confidence intervals (CIs) for ESRD according to the subgroups categorized by demographic and clinical factors were evaluated using the Cox proportional-hazards model. A p-value of less than 0.05 was considered statistically significant.

## Supplementary Information

Supplementary Information 1.

Supplementary Information 2.
