# Supplementary material for: The beneficial effects of menopausal hormone therapy on renal survival in postmenopausal Korean women from a nationwide health survey
Source: Sci Rep. 2021 Jul 29;11:15418. doi: 10.1038/s41598-021-93847-9 (PMC8322273; doi:10.1038/s41598-021-93847-9)
Supplement: Supplementary file 1 — Supplementary Information 1. [file 41598_2021_93847_MOESM1_ESM.docx]

**S1 Table. Hazard ratios and 95% confidence intervals of ESRD development by age at menopause**

| Total population | No. | Outcome | IR | Model 1* | Model 2† |
| --- | --- | --- | --- | --- | --- |
| **Age at menopause** | |  |  |  |  |
| < 50 yrs | 509,140 | 1,839 | 0.394 | 1.144 (1.077, 1.214) | 1.079 (1.016, 1.146) |
| 50 ≤ < 55 yrs | 795,070 | 2,519 | 0.344 | 1 (ref.) | 1 (ref.) |
| ≥ 55 yrs | 156,101 | 547 | 0.380 | 1.103 (1.006, 1.210) | 0.948 (0.864, 1.040) |

Abbreviations**:** ESRD, end stage renal disease; *Model 1: Not adjusted; †Model 2: Cox proportional model adjusted for age, BMI, smoking history, drinking history, degree of exercise, hypertension, dyslipidemia, diabetes, and CKD.
