# Supplementary material for: The beneficial effects of menopausal hormone therapy on renal survival in postmenopausal Korean women from a nationwide health survey
Source: Sci Rep. 2021 Jul 29;11:15418. doi: 10.1038/s41598-021-93847-9 (PMC8322273; doi:10.1038/s41598-021-93847-9)
Supplement: Supplementary file 2 — Supplementary Information 2. [file 41598_2021_93847_MOESM2_ESM.docx]

**Standardized questionnaire for breast cancer risk factors in the breast cancer screening program**

1. When did you start your menstruation?

① ____ years old

② I had no first period

2. What is your current menstruation status?

① I still have menstruations

② I underwent hysterectomy

③ I am menopause (Age of menopause: ____ years old

3. After menopause, to relieve symptoms, are you taking any hormone medications, or have you had them in the past?

① I never have had hormone medications

② I have had medication for less than 2 years

③ I have had medication for 2 years or more ~ less than 5 years

④ I have had medication for 5 years or longer

⑤ I do not know

4. How many children did you give birth to?

① One

② Two or more

③ None

5. Did you breastfeed and for totally how long?

① Less than 6 months

② 6 months ~less than 1 year

③ 1 year or longer

④ I have not breastfed before

6. Have you been diagnosed of benign breast tumors before? (Benign tumors mean tumors that are not cancer but are other cysts, lumps and etc)

① Yes

② No

③ I don’t know

7. Are you taking oral contraceptives or have you in the past?

① I have never taken oral contraceptives

② Less than 1 year

③ 1 year and longer

④ I don’t know
